# Early changes in muscle atrophy and muscle fiber type conversion after spinal cord transection and peripheral nerve transection in rats

**DOI:** 10.1186/1743-0003-10-46

**Published:** 2013-05-20

**Authors:** Kosaku Higashino, Tetsuya Matsuura, Katsuyoshi Suganuma, Kiminori Yukata, Toshihiko Nishisho, Natsuo Yasui

**Affiliations:** 1 Department of Orthopedics, Institute of Health Biosciences, The University of Tokushima Graduate School, 3-18-15, Kuramoto, Tokushima 770-8503, Japan

**Keywords:** Muscle atrophy, Muscle fiver type conversion, Spinal cord transection, Peripheral nerve transection, Rat

## Abstract

**Background:**

Spinal cord transection and peripheral nerve transection cause muscle atrophy and muscle fiber type conversion. It is still unknown how spinal cord transection and peripheral nerve transection each affect the differentiation of muscle fiber type conversion mechanism and muscle atrophy. The aim of our study was to evaluate the difference of muscle weight change, muscle fiber type conversion, and Peroxisome proliferator-activated receptor-γ coactivatior-1α (PGC-1α) expression brought about by spinal cord transection and by peripheral nerve transection.

**Methods:**

Twenty-four Wistar rats underwent surgery, the control rats underwent a laminectomy; the spinal cord injury group underwent a spinal cord transection; the denervation group underwent a sciatic nerve transection. The rats were harvested of the soleus muscle and the TA muscle at 0 week, 1 week and 2 weeks after surgery. Histological examination was assessed using hematoxylin and eosin (H&E) staining and immunofluorescent staing. Western blot was performed with 3 groups.

**Results:**

Both sciatic nerve transection and spinal cord transection caused muscle atrophy with the effect being more severe after sciatic nerve transection. Spinal cord transection caused a reduction in the expression of both sMHC protein and PGC-1α protein in the soleus muscle. On the other hand, sciatic nerve transection produced an increase in expression of sMHC protein and PGC-1α protein in the soleus muscle. The results of the expression of PGC-1α were expected in other words muscle atrophy after sciatic nerve transection is less than after spinal cord transection, however muscle atrophy after sciatic nerve transection was more severe than after spinal cord transection.

**Conclusion:**

In the conclusion, spinal cord transection diminished the expression of sMHC protein and PGC-1α protein in the soleus muscle. On the other hand, sciatic nerve transection enhanced the expression of sMHC protein and PGC-1α protein in the soleus muscle.

## Introduction

Nerve injury produces significant physiological and biochemical changes in skeletal muscle. These changes include loss of muscle mass, alteration in muscle fiber type, reduction in mitochondrial concentration, increase in resting blood flow, alteration in metabolite levels, and decrease resistance to fatigue [1-9].

Skeletal muscle contains two different types of muscle fiber: slow muscle fibers and fast muscle fibers [10,11]. It has been reported that spinal cord transection and denervation of peripheral nerve converts muscle fiber type from slow to fast phenotype in the soleus muscle [2,3,8,9,12-14]. The underlying mechanism of muscle fiber type conversion has not been well documented. However, recently it has been reported PGC-1α (peroxisome proliferator-activated receptor-γ coactivatior-1α) plays an important role in the conversion of fast muscle fiber into slow muscle fiber during skeletal muscle repair [15,16]. Peroxisome proliferator-activated receptor-γ coactivatior-1α (PGC-1α) is expressed in several tissues, including skeletal muscle and brown adipose tissue [16]. It has been shown that transcriptional co-activator PGC-1α increases the formation of slow muscle fibers [17,18]. PGC-1α is expressed in skeletal muscle, and induces mitochondrial biogenesis as well as activity of the myoglobin [15,16].

It is still not known how spinal cord transection and peripheral nerve transection each affect the differentiation of muscle fiber type conversion mechanism, muscle atrophy and PGC-1α expression. These findings led us to hypothesize that the degree of muscle atrophy is different between spinal cord injury and peripheral nerve injury, and PGC-1α expression is correlated to fiber type conversion after either of these injuries. The aim of this study was to evaluate the difference of muscle atrophy and fiber type conversion between after spinal cord injury and after peripheral nerve injury. Furthermore, we investigated the relationship between the muscle fiber type conversion and the PGC-1α expression in both injuries.

## Materials and methods

### Animal and experimental protocol

This study protocol conformed with the guidelines for the care and approved by a scientific board as well as the animal ethics committee of our University. After this approval twenty-four 8-week-old female Wistar rats, weighing 190.6 ± 3.9 g, were obtained from Charles River Japan Co., Ltd. (Yokohama, Japan). The rats were housed in individual cages and kept in a temperature and humidity controlled room (temperature 23 +/− 2C; humidity 55 +/− 10%) on a cycle consisting of 12 hours light and 12 hours dark. The rats had free access to food and water. The 24 rats were divided into three groups (n = 8): 1) the control rats underwent a laminectomy at T7-T8; 2) the rats in the spinal cord injury group underwent a laminectomy at T7-T8 followed by a spinal cord transection with a gap of 5 mm; 3) the rats in the denervation group underwent a sciatic nerve transection by removal of a sciatic nerve section 10 mm in length at both thighs.

The rats in the control group were euthanized at 0 week (n = 4), 1 week (n = 2) and 2 weeks (n = 2) after surgery. The rats in the spinal cord transection group were euthanized at 1 week (n = 4) and 2 weeks (n = 4) after surgery. The rats in the sciatic nerve transection group were euthanized at 1 week (n = 4) and 2 weeks (n = 4) after surgery. Each time a rat was euthanized its body weight was measured and then the soleus and tibialis anterior muscles were both harvested bilaterally. The muscle weight to body weight ratio was calculated. The soleus and tibialis anterior muscles were flash-frozen in 2-methylbutane pre-cooled in liquid nitrogen, and stored at −80 C pending histological analysis and Western blot analysis.

### Histological examination

Consecutive soleus and tibialis anterior muscle sections were cut in 5-μm-thick sections using the cryostat (Leica Microsystems AG, Wetzlar, Germany). Histological examination of muscle fiber atrophy was assessed using hematoxylin and eosin (H&E) staining. The diameters of the muscle fibers were measured using computerized interactive software (Win ROOF, Mitani Co. Fukui & Tokyo, Japan); at least 100 fibers per specimen were counted (magnification × 200) (Mitani Co. Fukui & Tokyo, Japan).

For immunofluorescent staining 5-μm thick sections were cut and washed 3 times in PBS. The sections were fixed with 4% paraformaldehyde for 5 minutes. They were again washed 2 times and then blocked with normal horse serum for 1 hour. The sections were incubated with monoclonal anti-slow myosin heavy chain (sMHC) antibody (dilution 1:200; Sigma, St. Louis, MO) for 1 hour. After being washed 3 times, the sections were incubated with anti-mouse IgG-Cy3 conjugate antibody (dilution 1:250; Sigma, St. Louis, MO). After being washed 3 times the sections were viewed under the fluorescent microscope to visualize the immunofluorescent results (Nikon, E600 and TE2000U, Tokyo, Japan). For the measurement of the muscle fiber type conversion, the sMHC muscle fibers (stained red) were measured on the cross-sectional area using computerized interactive software (Win ROOF, Mitani Co. Fukui & Tokyo, Japan).

### Western blot analysis

Muscle samples were homogenized and lysed with RIPA buffer (Tris–HCl, NaCl, 1% TritonX-100, EDTA · 2Na · H_2_0). The protein concentration on each sample was determined using the Lowry method [19]. Western blot was performed with total tissue homogenates on 7% and 10% polyacrylmamide gels after the protein content was quantified. A total of 30 μg protein was applied on each lane of the gels. The following antibodies were used; anti-PGC-1 (dilution 1:1000; AB3242, Chemicon, Temecula, CA), NOQ7.5.4D, monoclonal mouse anti-slow myosin heavy chain (sMHC) antibody (1:1000; Sigma, St. Louis, MO). Anti-β-actin (diluted 1:1000; Sigma) was used for protein quantification.

The statistical difference was determined by Mann-Whitney’s *U* test. All statistical analyses were performed using StatView, version 5.0 (Abacus Concepts, Berkeley, CA), with P < 0.05 considered statistically significant.

## Results

**Figure 1:**
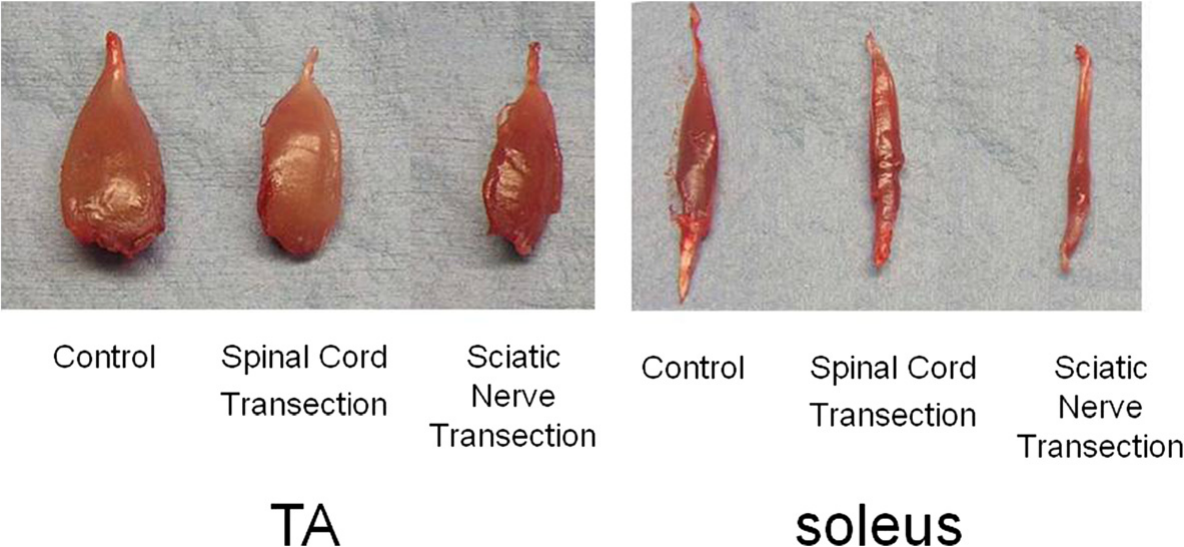
Two weeks after surgery, macroscopic anatomy of both tibialis anterior muscle (TA) and soleus muscle showed the atrophy of the muscle in the sciatic nerve transection group was severe than that in spinal cord transection group.

Two weeks after surgery, the body weights of the rats that had received spinal cord transection were significantly less than the body weights of the rats that had received sciatic nerve transection. Spinal cord transection and sciatic nerve transection caused severe muscle weight loss for both the soleus and the tibialis anterior muscles. The muscle weight and the muscle weight to body weight ratio after sciatic nerve transection were both significantly less than those after spinal cord transection (Table 1, Figure 1).

**Table 1:** Initial and final body weight and muscle weight

	**0w**	**Control 2w**	**SCT 2w**	**SNT 2w**
Body weight (g),	190.6 ± 3.9	222.5 ± 4.6†	209.1 ± 2.6 †‡	222.1 ± 5.2 ‡
TA (mg)	35.1 ± 2.8	43.1 ± 3.2†	30.0 ± 5.6 †‡	21.4 ± 2.4 ‡
TA/body weight (mg/g)	18.4	19.4†	14.8 †‡	9.6 ‡
Soleus (mg)	108.8 ± 5.6	123.2 ± 9.3†	77.3 ± 2.7 †‡	44.6 ± 4.6 ‡
Soleus/body weight (mg/g)	0.57	0.55†	0.037 †‡	0.020 ‡

†‡ Mann–Whitney *U* test, alpha = 0.05, †‡ Significant difference.

**Figure 2:**
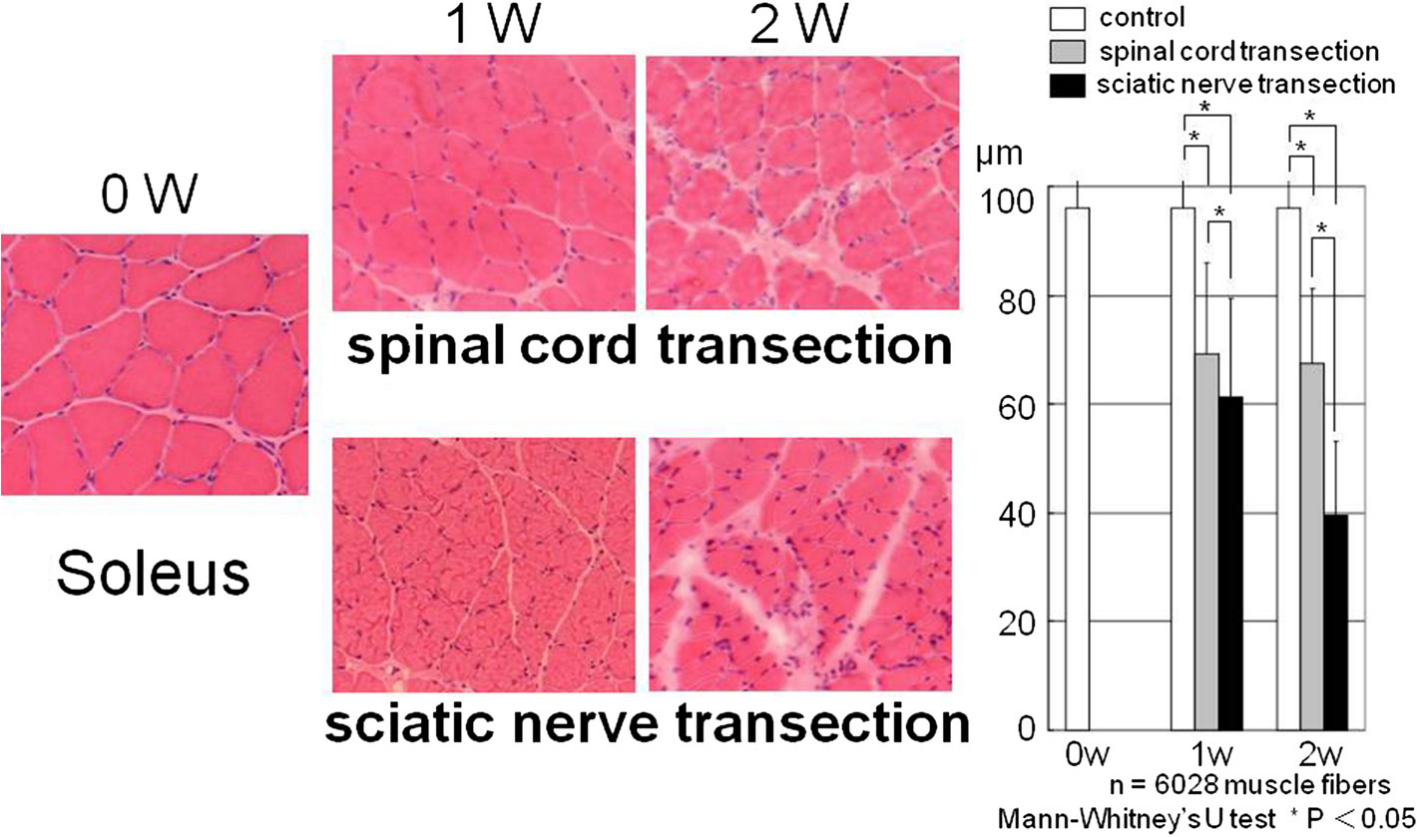
**The mean diameter of the soleus muscle fiber in the sciatic nerve transection group was significantly less than that in the spinal cord tarnsection group.** The open bars indicate the control at 0 week, 1 week and 2 weeks, the gray bars indicate 1 week and 2 weeks after spinal cord transection, and the black bars indicate 1 week and 2 weeks after sciatic nerve transection.

In the control group, the mean diameter of the soleus muscle fiber was 96.3 ± 22.5 μm at 0 week, 96.0 ± 16.8 μm at 1 week, and 96.3 ± 13.6 μm at 2 weeks. In the spinal cord transection group, the mean diameter of the soleus muscle fiber was 69.1 ± 16.8 μm at 1 week after surgery, and 67.4 ± 13.6 μm at 2 weeks after surgery. In the sciatic nerve transection group, the mean diameter on the soleus muscle fiber was 61.4 ± 17.9 μm at 1 week after surgery and 39.4 ± 13.5 μm at 2 weeks after surgery. The mean diameter of the soleus muscle fiber was significantly less in the sciatic nerve transection group than in the spinal cord transection group at both time points (Figure 2).

**Figure 3:**
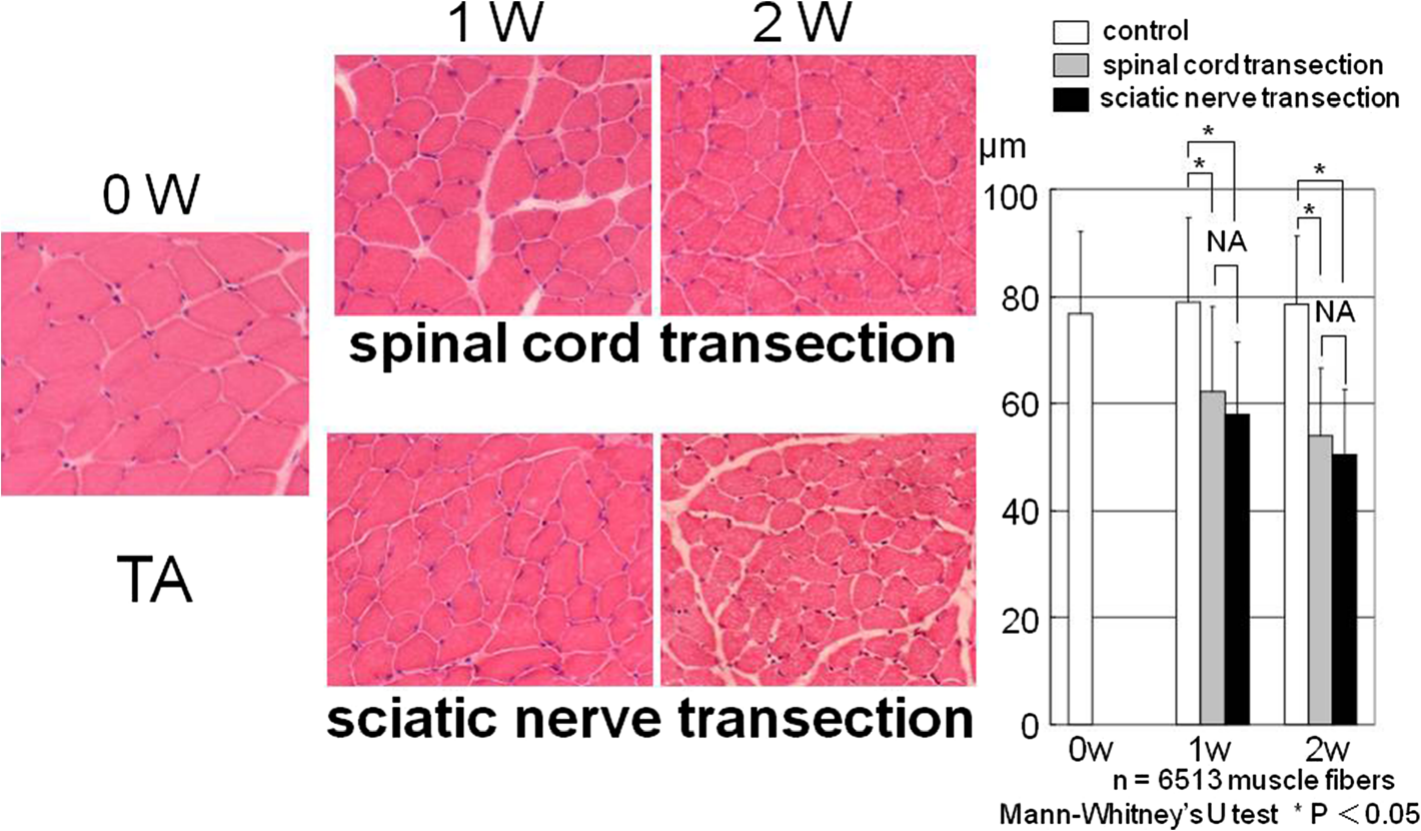
**The mean diameters of the tibalis anterior muscle fiber in the spinal cord transection group and in the sciatic nerve transection group were less than those in the control group.** There was no significant difference between the mean diameter of tibialis anterior muscle fiber in the spinal cord transection group and in the sciatic nerve transaction group. The open bars indicate control at 0 week, 1 week and 2 weeks, the gray bars indicate 1 and 2 weeks after spinal cord transection, and the black bars indicate 1 and 2 weeks after sciatic nerve transection.

In the control group, the mean diameter of the tibialis anterior muscle fiber was 76.9 ± 15.4 μm at 0 week, 78.9 ± 15.7 μm at 1 week, and 78.5 ± 12.5 μm at 2 weeks. In the spinal cord transection group the mean diameter of the tibalis anterior muscle fiber was 62.2 ± 15.8 μm at 1 week after surgery, and 54.0 ± 12.6 μm at 2 weeks after surgery. In the sciatic nerve transection group the mean diameter of the tibialis anterior muscle fiber was 57.7 ± 13.7 μm at 1 week, after surgery and 50.5 ± 12.1 μm at 2 weeks after surgery. There were no significant differences at either time point in the mean diameter of tibialis anterior muscle fibers between the sciatic nerve transection group and the spinal cord transection group (Figure 3).

**Figure 4:**
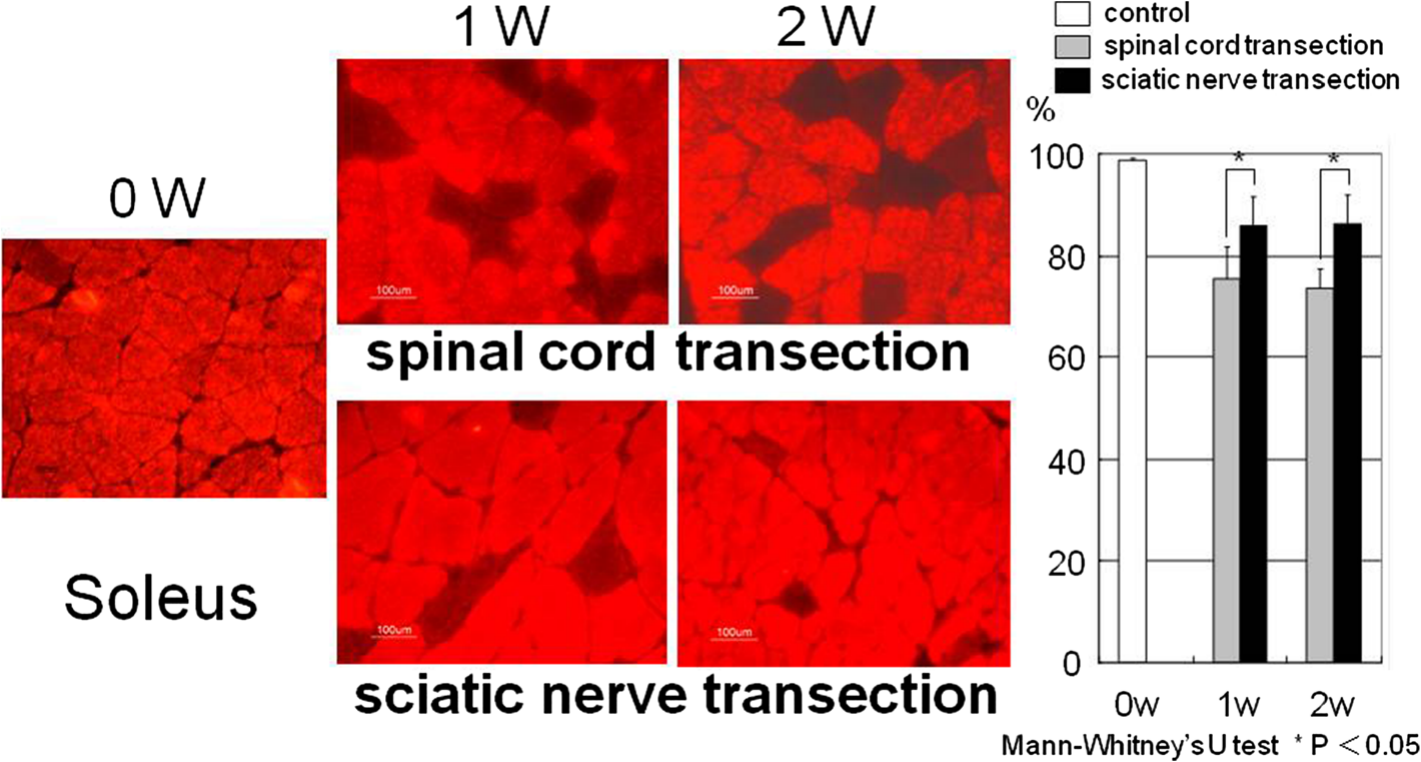
**Immunofluorescent staining for the slow myosin heavy chain (sMHC).** The red muscle fibers on the cross-sectional area were positive for sMHC antibody. For the soleus muscle the percentage of positive area in the spinal cord transection group was significantly less than that in the sciatic nerve transection group.

In the control group at 0 week for the soleus muscle the percentage of the area stained sMHC antibody showed 98.8% ± 0.2% in the cross-sectional area. In the spinal cord transection group, for the soleus muscle the percentage of the area stained sMHC antibody was 75.6% ± 6.2% at 1 week after surgery and 73.6% ± 3.8% at 2 weeks after surgery. In the sciatic nerve transection group, for the soleus muscle the percentage of the area stained sMHC antibody was 86.0% ± 5.4% at 1 week after surgery and 86.0% ± 5.7% at 2 weeks after surgery. The percentage of the area stained sMHC antibody was significantly greater in the sciatic nerve transection group than in the spinal cord transection group at both time points (Figure 4).

**Figure 5:**
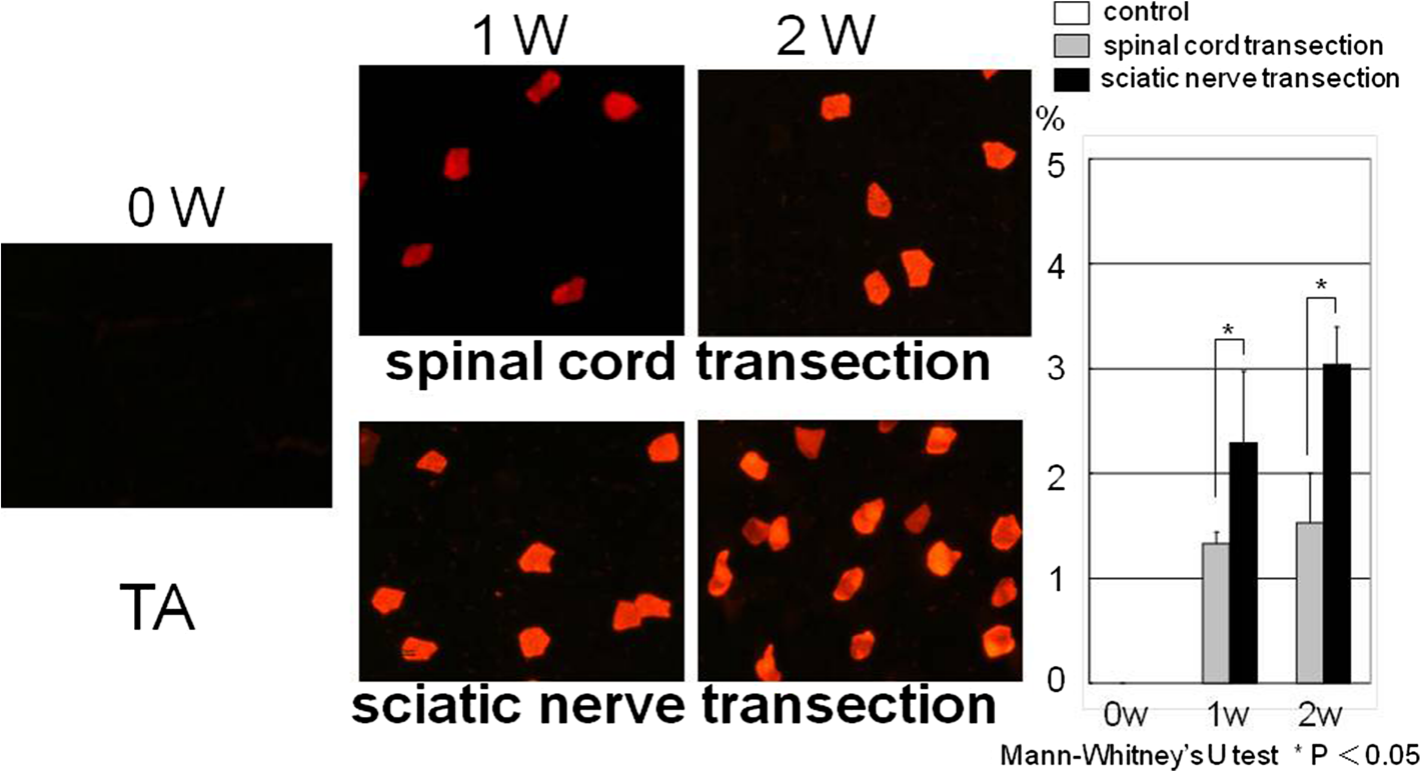
**In the control group, the tibialis anterior superficial (TA) muscle fibers were negative for staining of slow myosin heavy chain (sMHC) antibody.** The percentage of the positive area at 1 week and 2 weeks in the sciatic nerve transection group was significantly greater than that in the spinal cord transection group.

In the control group at 0 week, for the tibialis anterior muscle superficial area no part of the cross sectional area stained sMHC antibody. In the spinal cord transection group, for the tibialis anterior muscle superficial area the percentage of the area that stained sMHC antibody was 1.3% ± 0.1% at 1 week after surgery and 1.5% ± 0.4% at 2 weeks after surgery. In the sciatic nerve transection group, for the tibialis anterior muscle superficial area the percentage of the area that stained sMHC antibody was 2.3% ± 0.7% at 1 week after surgery and 3.1% ± 0.3% at 2 weeks after surgery. The percentage of the area stained sMHC antibody was significantly greater in the sciatic nerve transection group than in the spinal cord transection group at both time points (Figure 5).

**Figure 6:**
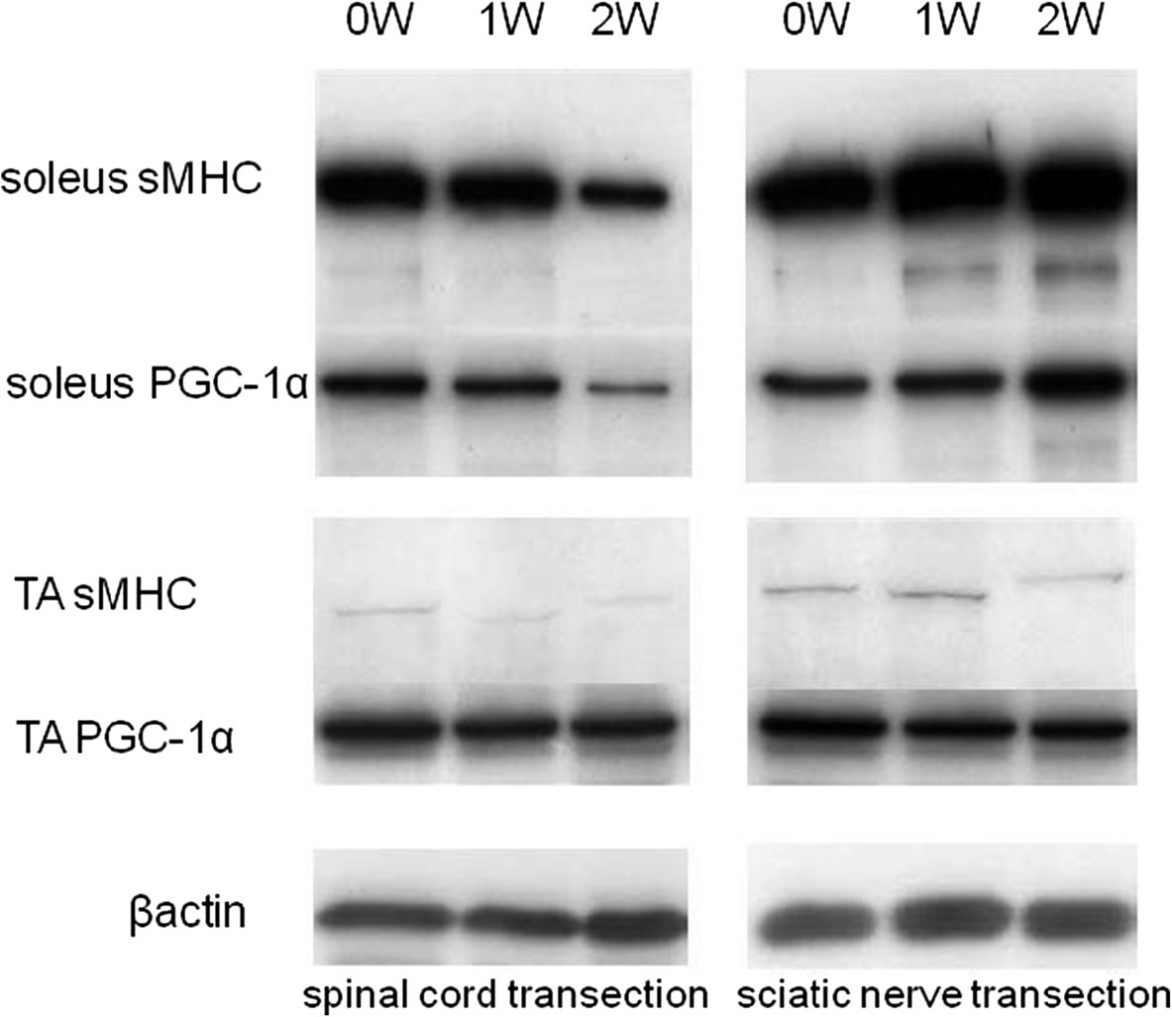
**Western blot pattern showed the expression of sMHC (slow myosin heavy chain) in the soleus muscle decreased with time after spinal cord transection, whereas increased with time after sciatic nerve transection.** For the tibialis anterior muscle, there was no observed expression of the proteins of sMHC and PGC-1 (peroxisome proliferator-activated receptor-γ coactivatior-1α) in the spinal cord transection group and in the sciatic nerve transection group. β actin, as a control.

In the sciatic nerve transection group, the expression of sMHC protein in the soleus muscle was greater than that in the control group at 1 and 2 weeks. The expression of PGC-1 protein was also greater in relation to the expression of sMHC protein in the soleus muscle after sciatic nerve transection. On the other hand, in the spinal cord transection group, the expression of sMHC protein in the soleus muscle was less than that in the control group. The expression of PGC-1 protein was also decreased in the soleus muscle after spinal cord transection (Figure 6).

## Discussion

The main results to emerge from this experiment are: 1) Both sciatic nerve transection and spinal cord transection caused muscle atrophy with the effect being more severe after sciatic nerve transection. 2) Spinal cord transection reduced the percentage of the area stained sMHC antibody in the soleus muscle. Sciatic nerve transection did not reduce the percentage of the area stained sMHC antibody in the soleus muscle strongly. For the tibialis anterior muscle sciatic nerve transection increased the percentage of the area stained sMHC antibody greater than the spinal cord transection. 3) Spinal cord transection caused a reduction in the expression of both sMHC protein and PGC-1α protein in the soleus muscle. On the other hand, sciatic nerve transection produced an increase in expression of sMHC protein and PGC-1α protein in the soleus muscle.

At 2 weeks after surgery the body weight of the rats in the sciatic nerve transection group was greater than that of the rats in the spinal cord transection group. For the soleus muscle, the atrophy of the muscle fiber as well as the muscle weight loss were both greater in the sciatic nerve transection group as compared to that in spinal cord transection group. Thus, although the body weight of the rats in the sciatic nerve group was maintained, indicating good general nutrition, this was not reflected in muscle weight, which decreased.

For both the soleus muscle and tibialis anterior muscle the percentage of slow muscle fiber was significantly greater in the sciatic nerve transection group than in the spinal cord transection group. These results indicate that spinal cord transection reduced slow muscle fiber type. On the other hand, sciatic nerve transection did not reduce slow muscle fiber type strongly.

Our results show that the soleus muscle contains 98% slow type muscle fibers and is strongly affected by sciatic nerve transection. In the spinal cord transection group, our immunofluorescent results and Western blotting results show that sMHC- expressing muscle fibers in the soleus muscle decreased with time. For the soleus muscle, the protein level of PGC-1α also showed a decrease with time in the spinal cord transection group. The expression of PGC-1α was abundant in skeletal muscle, particularly in slow fiber types. PGC-1α induces the production of mitochondria and synthesis of slow contractile proteins [16]. It was reported that PGC-1α activation plays an important role in up-regulating the formation of slow muscle fibers [17,18]. Slow muscle fibers expression and PGC-1α protects against muscle atrophy due to the inhibition of atrogenes [18,20].

Our results for the tibialis anterior muscle after sciatic nerve transection show a relationship between the expression of sMHC and the increase in expression of PGC-1α. The results of the expression of PGC-1α were expected in other words muscle atrophy after sciatic nerve transection is less than after spinal cord transection, however muscle atrophy after sciatic nerve transection was more severe than after spinal cord transection.

The difference between spinal cord injury and peripheral injury is survival of the γ-loop pathway [21,22]. Destruction of the γ-loop pathway causes severe muscle atrophy and induced slow muscle fiber type conversion in fast muscle fiber muscle [23].

Future studies will need to be carried out to investigate the relationship between skeletal muscle atrophy and fiber type conversion after spinal cord transection and sciatic nerve transection.

## Competing interests

The authors declare that they have no competing interests.

## Authors’ contributions

KH carried out the all histologic and molecular studies, participated in the drafted the manuscript. TM carried out the immunohistochemistry and helped to draft the manuscripts. KS carried out histologic analysis. KY participated in the design of study. TN participated in the molecular studies. NY participated in the coordination of the manuscripts and helped to draft the manuscripts. All authors read and approved the final manuscript.
